# Ruminal Acidosis in Feedlot: From Aetiology to Prevention

**DOI:** 10.1155/2014/702572

**Published:** 2014-11-12

**Authors:** Joaquín Hernández, José Luis Benedito, Angel Abuelo, Cristina Castillo

**Affiliations:** Department of Animal Pathology, Faculty of Veterinary Science, University of Santiago de Compostela, Campus Universitario, 27002 Lugo, Spain

## Abstract

Acute ruminal acidosis is a metabolic status defined by decreased blood pH and bicarbonate, caused by overproduction of ruminal D-lactate. It will appear when animals ingest excessive amount of nonstructural carbohydrates with low neutral detergent fiber. Animals will show ruminal hypotony/atony with hydrorumen and a typical parakeratosis-rumenitis liver abscess complex, associated with a plethora of systemic manifestations such as diarrhea and dehydration, liver abscesses, infections of the lung, the heart, and/or the kidney, and laminitis, as well as neurologic symptoms due to both cerebrocortical necrosis and the direct effect of D-lactate on neurons. In feedlots, warning signs include decrease in chewing activity, weight, and dry matter intake and increase in laminitis and diarrhea prevalence. The prognosis is quite variable. Treatment will be based on the control of systemic acidosis and dehydration. Prevention is the most important tool and will require normalization of ruminal pH and microbiota. Appropriate feeding strategies are essential and involve changing the dietary composition to increase neutral detergent fiber content and greater particle size and length. Appropriate grain processing can control the fermentation rate while additives such as prebiotics or probiotics can help to stabilize the ruminal environment. Immunization against producers of D-lactate is being explored.

## 1. Introduction

Grain overload in feedlot cattle has gained attention because of its economic impact. Economics of feedlot beef production dictate that cattle must gain weight at their maximum potential rate; this involves getting them quickly onto a full feed of a diet containing a high concentration of grain. Economics also favour processing of grain by available methods to increase the digestibility of starch. All of these factors set the stage for grain overload in feedlot cattle [1, 2]. In fact, digestive disorders, including ruminal acidosis, are second only to respiratory diseases in depressing animal performance and production efficiency [3]. However, there is still a lack of data from the field and, moreover, of uniformity in definition, and clinical diagnosis terminology varies and different descriptions of the disease are made [4]. Therefore, it appears to be useful to review the literature on this topic.

This paper provides an overview of research of this digestive disease in beef cattle, with different preventive measures and nutritional alternatives.

## 2. What Is Ruminal Acidosis and How Is It Manifested?

Ruminal acidosis is frequently defined as a decrease in the ruminal pH. But the question is whether this condition is a disease or not. So, many researchers recognized that acidosis is not one disease, but rather a continuum of degrees of ruminal acidity, because nonphysiological accumulation of organic acids and consequent reduction in pH below the normal have a significant impact on microbial activity, rumen function, and animal productivity and health [5].

Accordingly, it may be better to define ruminal acidosis as a fermentation disorder in the rumen characterized by a lower than normal ruminal pH, but reflecting an imbalance between microbial production, microbial utilization, and ruminal absorption of volatile fatty acids (VFA) [6]. Some 30% to 50% of the acid in the rumen is neutralized by salivary buffers or bound to ammonia generated from urea entering across the ruminal wall. A smaller quantity passes on into the lower gastrointestinal tract [7, 8]. However, even the most conservative estimates leave a significant proportion of about 30–50% of the acid that is ruminally produced and that has to be absorbed by the ruminal wall, and one of the most important reasons for the appearance of ruminal acidosis would be a decrease in the absorptive capacity of the rumen which is thus unable to maintain a stable pH.

Absorption of VFA, by removing unionized acid and by the exchange of ionized VFA for bicarbonate during the absorption process, aids in maintaining pH near neutrality. Consequently, a reduced rate of VFA absorption causes ruminal pH to drop for two reasons: ruminal VFA accumulate and bicarbonate input from the blood stream is decreased [1].

The severity of acidosis allows us to classify ruminal acidosis considering different factors, among others, like ruminal pH threshold, predominant acid (VFA or lactic), and ruminal population bacteria, in two forms: acute and subacute acidosis (see Table 1).

In a brief summary, and starting with clinical signs, in acute forms symptoms will appear in the animal, more or less noticeable, and will be absent in a subacute form. Taking into account ruminal parameters, ruminal pH will be low in acute form, and this fact will imply an important difference in bacterial species, with gram negative bacteria appearing, with lactate consumers bacteria, and high amount of VFA. Meanwhile, in an acute form, we will find gram positive bacteria, with lactate producer bacteria, like* Streptococcus bovis* or even, in ruminal pH below than 4.8,* Lactobacillus *spp. In this severe form, with pH next to isoelectric point of lactic acid (around 3.8), we will find metabolic acidosis, with a decrease in blood pH and blood bicarbonate, increasing the amount of serum lactate and decreasing the presence of lactate in the rumen.

In conclusion, we can define* acute ruminal acidosis* as a metabolic status defined by a decrease in blood pH, parallel to blood bicarbonate decrease, which is caused by a D-lactic ruminal overproduction.

## 3. Is Lactic Acid Appropriate for the Animals or Is It Toxic to Them?

In reality, this acid is useful for animals, because lactate is an important electron donor for sequential nitrate reduction to ammonia in the rumen. What is more important is that lactate can serve as an effective electron donor for nitrite reduction, which implies that “nitrate toxicity” may be prevented by an adequate supply of lactate when ruminants ingest diets containing high levels of nitrate. Moreover, augmentation of nitrate reduction to ammonia potentially reduces ruminal methanogenesis, as previously reported. In this regard, lactate production is beneficial for the overall ruminal fermentation.

**Figure 1:**
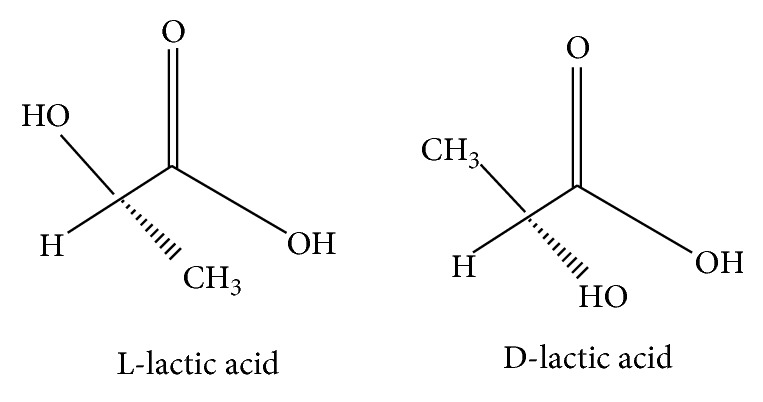
Optical forms of lactate: L(+) and D(−) lactate. The sign (−) is because the molecule makes polarized light turn left (*levorotatory lactic acid*); the opposite for the (+) sign (*dextrorotatory lactic acid*).

In relation to lactic acid, also called hydroxypropanoic acid, it was discovered by Scheele in 1870, who isolated the lactic acid from sour milk as an impure brown syrup and gave it a name based on its origins. Lactic acid has two optical forms, L(+) and D(−), and there is often a marked difference in the effects of two enantiomers on living beings, although stereoisomers have similar physical and chemical properties (Figure 1).

An important difference between both molecules gives reference to its particular metabolization and elimination. So, L-lactate is quickly metabolized in the liver, by* L-lactic dehydrogenase* (EC 1.1.1.27), to pyruvate, which will be turned into glucose through the gluconeogenesis. In relation to D-lactate, this molecule is metabolized by* D-*α*-hydroxy acid dehydrogenase* (EC 1.1.99.6) and by* D-lactate dehydrogenase* (EC 1.1.1.28); the latter is able to convert pyruvate to D-lactate. Another important difference between them is their renal excretion capacity, higher for L-lactate, at least at the beginning, although both enantiomers share the same zone for renal excretion. When serum value of D-lactate is increased, it will increase their excretion ratio, exchanging with L-lactate [8].

In cows, lactic acidosis has been related to different diseases in young calves (suckling calves or growing calves) and in mature cows. In the latter, acid is produced in rumen or in gastrointestinal system, like humans, because they contain the same transporter, called* proton-dependent monocarboxylate*; members of the SLC16A family are proton-linked transporters that play a crucial role in cellular metabolism. These isoforms differ in terms of tissue distribution, substrate specifics, and affinities with only four isoforms (MCT1-4) characterized as proton-dependent monocarboxylate transporters, being the most important for D-lactate two isoforms (MCT-1 and MCT-2), especially isoform 1, from intestine to the bloodstream, allowing the acid metabolization in the cytosol of the liver or heart cells. Some similar processes with high levels of D-lactate have been described in other animals, like cats with pancreatic failure, kids (floppy kid syndrome), or lambs (drunken lamb syndrome) and even humans (short-bowel syndrome, colorectal cancer).

## 4. Which Is the Origin of This Disease?

There are three main groups of reasons that, isolated from combining, can produce ruminal acidosis. They are summarized in Table 2.

In the last year, new focus has been directed towards the feeding behaviour, due to the fact that dry matter intake determines the acid production, and the chewing activity determines the buffer capacity, and both, together, determine the ruminal pH. But it is important to remark that susceptibility to suffer from this problem is individual, so animals in the same barn will not necessarily suffer from the same probability of developing this problem, possibly related to hierarchy or dominance patterns.

Calsamiglia et al. [9] coined the name of* concentrate syndrome* for this problem because this process is related to two different facts: (i) decrease in ruminal pH and (ii) changes in the ruminal microbiota population and both are responsible for the process, in a combined way. This point of view is very interesting in order to apply some preventive measures, as we will see later.

An interesting fact was pointed out in other studies [10] talking about nutritional synchrony, which presumes that the diet is the major determinant of the quantity and quality of nutrients supplied to the rumen microbial population and to the animal. In reality, multiple ruminal and endogenous pools determine nutrient availability to the rumen and animal. Factors like immune status, damage to tissue function, and the animal's own metabolic fluctuations may alter response to the diet. The risk for acidosis is not equal for all animals, and, presumably, it is related to the combined effects of level of feed intake, eating rate, sorting of feed, salivation rate, the inherent ruminal microbial population, previous exposure to acidosis, rate of passage of feed from the rumen, and other aspects of physiology and behavior [11].

**Figure 2:**
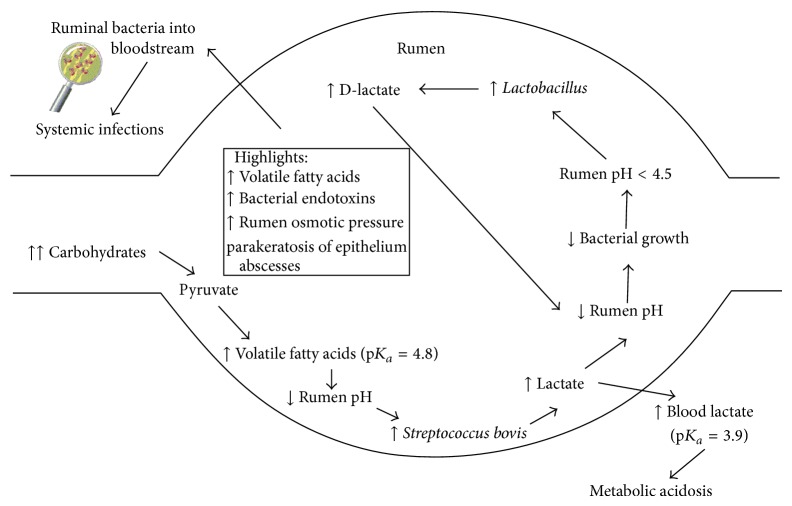
Cascade of events in ruminal acidosis.

After the consumption of a high grain diet, nonstructural carbohydrates will arrive to the rumen (physiological process), promoting their fermentation by amylolytic bacteria, producing pyruvate and finally volatile fatty acids (VFA), dissociating, and producing a drop in ruminal pH. This drop implies that many gram (−) bacteria disappear, including lactate-consuming bacteria, like* Megasphaera elsdenii* and* Selenomonas ruminantium* (convert lactate to pyruvate), because they are sensitive to pH. Conversely, there is an increase in the population of some gram (+) bacteria, especially Streptococcus bovis, known as a lactate-producing bacteria; thereby promoting a second ruminal bacterial population change, due to a new drop in ruminal pH, derived from increase in L-lactic acid, which is a very potent acid (10 times stronger than VFA), and this property contributes further to the decline in ruminal pH, growing only bacterial pH resistance, like* Lactobacilli *spp., great lactate producer bacteria, especially for D-lactate, which will conduct a new drop of ruminal pH, up to 3.8, an isoelectric point for this acid, and, in this moment, acid will be undissociated, crossing the ruminal wall to the bloodstream and provoking a metabolic acidosis (Figure 2).

## 5. Clinical Picture of the Disease (Figure 3)

The onset of the clinical signs associated with ruminal acidosis will depend on the clinical form, varying from sudden death in peracute course to a light feed depression in subacute way. It is normal to talk about the relationship between ruminal acidosis and ruminal hypotony or even atony, producing this last one by some different, and not excluded, mechanism.

(1) Direct action of the VFA is one of the most important mechanisms to consider, since chemical receptors in the epithelium send a feedback signal to the brain to reduce ruminal motility. When VFA contacts with them, signal will be sent to the central nervous system, promoting ruminal atony [7].

(2) Another mechanism to promote the rumen hypomotility is related to the increase in the osmolality in the ruminal content, produced by the accumulation of organic acids and glucose increasing the osmotic pressure inside the rumen, which implies a water flux from the bloodstream across the rumen wall, sometimes producing a* hydrorumen*. As a consequence of* hydrorumen*, animal will show a decrease in packed cellular volume, with haemoconcentration and sometimes polyuria, with the animal feeling dehydrated. Taking into account an abnormal composition of the ruminal juice, animal may show diarrhea, which will complicate the hydroelectrolytic balance of the animal. Considering that the structure and consistency of the faeces depend on rumination, activity of the ruminal flora and ruminal passage, the animal will show some changes in colour, odour, pH, and consistency, and even whole cereal grains may be present. The impaired ruminal function in terms of rumination, bacterial breakdown, and passage leads to the alteration in faecal aspects [4, 12].

(3) The third mechanism involved in this hypomotility is the role of the different vasoactive substances, such as histamine, tyramine, and tryptamine, which are produced in the rumen by decarboxylation of histidine, tyrosine, and tryptophan, respectively. Bacterial endotoxins have been related to the decrease in rumen motility although the exact mechanism remains unclear [4, 12].

The growth of ruminal epithelium has been shown to be directly linked to the nonstructural carbohydrates presence in the tissue. Propionic and butyric acid are promoting the growth of the ruminal papillae, thus providing a higher absorption from the rumen by the mucosa, but, in a low ruminal pH, with excessive amount of VFA, will lead to a parakeratosis of the ruminal epithelium, and this parakeratosis will lead to rumenitis, particularly the presence of microabscesses within the ruminal mucosa, favouring to incorporate with the bloodstream of the different ruminal bacteria, especially among others, with* Fusobacterium necrophorum* and* Arcanobacterium pyogenes*, colonizing the liver tissue and from there spreading to other organs like kidneys, heart, and lungs [13, 14] and promoting the* parakeratosis-rumenitis liver abscesses complex* [13].

One important complication is that, as a consequence of the ruminal mucosae destruction, many anaerobic bacteria will be able to cross the ruminal wall, incorporating with the bloodstream and favouring infections like pneumonia, pyelonephritis, and typical endocarditis. In fact, we could be able to isolate* Arcanobacterium pyogenes* inside the valve, a typical ruminal bacterium.

Another symptom is that the animal would develop polioencephalomalacia, produced by a B1 vitamin or thiamine deficit. The bacteria in the rumen normally create this vitamin, so cattle do not normally need it in feed. So, thiamine inadequacy can be caused by decreased production by rumen microbes or factors that interfere with the action of thiamine, for example, plant thiaminases or thiamine analogs. Thiaminases can be produced by gut bacteria or ingested as preformed plant products. They can either destroy thiamine or form antimetabolites that interfere with thiamine function.* Thiaminase I*, produced by* Bacillus thiaminolyticus* and* Clostridium sporogenes*, and* thiaminase II*, produced by* Bacillus aneurinolyticus*, catalyze the cleavage of thiamine. The latter microorganism proliferates under conditions of high grain intake. Neurologic symptoms include depression, anorexia, blindness, convulsions, incoordination, depression, and opisthotonos in standing position, and even animals show a typical* star grazing stand*. It is important to point out that many of the neurologic signs are not promoted by thiamine deficit, because the serum D-lactic acid increase allows it to cross the blood-brain barrier by monocarboxylate protons transporters. The majority of neurological disturbances (i.e., ataxia and depressed menace, palpebral, and tactile reflexes) are related to D-lactate accumulation in cerebrospinal fluids rather than in blood [15].

One clinical sign regularly mentioned to be associated with ruminal acidosis is laminitis [4], or* pododermatitis aseptica diffusa*, which is an aseptic inflammation of the dermal layers inside the foot. Nutritional management has been identified as a key component in the development of laminitis, particularly the feeding of increased fermentable carbohydrate, which results in an acidotic state. It is suspected that there are vasoactive substances entering the bloodstream from the rumen, leading to damage in the corium. The initial insult is thought to be metabolic in nature like a low ruminal pH. This allows a chain of pathological mechanisms to take place, eventually leading to ischemia of the distal limb and a clinically detectable form of laminitis, manifesting by blood imbibition of the sole during acute phases of the disease and classical picture of hoof deformation as the disease becomes chronic. Histamine, lipopolysaccharide endotoxin (LPS) [8], and lactate are biological active agents suspected to interact in this complex [12], although it is true that the most important histamine producer,* Allisonella histaminiformans*, increases at alkaline pH, although it is able to grow at ruminal pH around 4.5. This fact implies that, probably, the main reason for the relationship between acidosis and laminitis will be not only an increase in the histamine production, but also a decrease in the histamine destruction, because, at low pH, there is a decreased diamine oxidase activity, promoting a histamine increase net flux from rumen to bloodstream. The role of tyramine and tryptamine, other vasoactive substances related to vascular episodes in the corium of the hoof and produced from tyrosine and tryptophan, respectively, remains unclear at this moment in the pathogenesis of the process. Bacterial endotoxins present in ruminal fluid also have been named as a possibly causative agent in the bovine laminitis complex. In an acidotic environment, the ruminal flora changes to a mainly gram-positive pattern. It has been shown that there is a detectable increase in endotoxins in the rumen, probably derived from the breakdown of the gram-negative bacteria [4], that damage the capillaries of the lamellae in the foot and cause hemorrhage, inflammation, and lameness [8], albeit it has been demonstrated that grain-induced SARA increased free LPS in the rumen but not in peripheral blood, which disagrees with the hypothesis that LPS damages the capillaries of the hoof [16].

Finally, as a consequence of the metabolic acidosis, animal could show symptoms like hyperventilation and signs derived from compensatory hyperkalemia. Note that hyperkalemia could develop from itself ventricular fibrillation or cardiac arrest, producing the death of the animal in some circumstances.

### 5.1. Changes in Blood Parameters

Blood analysis will show a leukocytosis with neutrophilia, derived from stress, and anemia and decrease in the packed cell volume, due to ruminal ulcers and hyporexia. In acid-base parameters, blood pH, base excess, and bicarbonate will be low, with an increase in anion gap, because lactate will act as an unmeasurable anion, decreasing measurable anion, in this case bicarbonate, in order to guarantee the electroneutrality principle (Table 1).

### 5.2. Changes in Ruminal Fluid

It is very important to consider the way for acquisition using probes, because contamination with saliva has to be accepted, although a discard of the first portion collected may decrease the influence, around 0.14 to 0.19 basic points [12]. Perhaps a good alternative to avoid this problem could be a transcutaneous puncture, or rumenocentesis, obtaining a ruminal pH value in this technique on average 0.37 units lower than in the samples collected through the probe, although some complications have been described for this puncture. Some researchers [17] observed that the pH of rumen fluid samples using a stomach tube (ororuminal probe) which are collected from the ventral sac of the rumen through a cannula were on average 0.35 and 0.33 pH units higher than the pH of rumen fluid samples collected by rumenocentesis.

More ruminal interesting parameters could be physical characteristics like colour (white), smell (acid, not aromatic), and consistency. From a microbiological point of view (see Table 1), in acidosis we will find an increased gram-positive bacteria population, as they are resistant to a low pH environment; whereas the population of gram-negative bacteria is decreased or absent. The lactate-producing bacteria* Streptococcus bovis* increases in acute ruminal acidosis, while lactate-utilizing species decrease. With a decrease in lactate-utilizing bacteria, lactate accumulates in the rumen during acute lactic acidosis. This will contribute further to a decreased or complete defaunation of ciliated protozoa in a low ruminal pH environment; and if we execute the methylene blue reduction test (also called methylene blue decolourisation test), we will see a decrease in the time necessary to convert the blue color to white colour, as an index of redox potential (remember, in acidosis, redox potential could be increased at the beginning of the process).

## 6. How to Manage It? The Role of Prevention

Individual cattle can be treated successfully, although the chances of success depend on the severity of the case [18], based on controlling changes associated with systemic acidosis and dehydration (we will apply fluid therapy, avoiding lactate enrichment fluids, such as Ringer lactate), and trying to correct complications, trying to restabilize ruminal functions.

In the herd, the most important thing to do is to anticipate the ruminal acidosis, and in order to do that the Reference Advisory Group on Fermentative Acidosis of Ruminants (RAGFAR) [18] have proposed some indirect indicators of ruminal acidosis in feedlot cattle; among others are

- decline in pen feed consumption of more than 10% for two or more consecutive days, causing a weight loss,
- a pen incidence of bubbly scours of more than 3% on any given pen inspection,
- evidence of laminitis in any* Bos taurus* cattle and more than 3% of* Bos indicus* cattle,
- a decrease in chewing activity (less than 50% of the calf rest time), due to a decrease in neutral detergent fiber.

But prevention is the most important tool to avoid acidosis appearing. In order to do that, we would keep the ruminal pH in physiologic ranges, increasing the neutral detergent fiber and decreasing concentrate intake and, in a second place, trying to keep ruminal microbiota, which will allow controlling the fermentative process. There are three strategies for the prevention of the high-concentrate syndrome [9]: (1) proper diet balancing and feeding management, (2) control of ruminal pH, and (3) control of the fermentation process.

### 6.1. Feeding Management Strategies

Feeding management includes changes to diet composition, increasing fiber content, and applying feed additives [7].

Applying diet changes includes giving the animals a proper balanced diet, increasing the FND to stimulate the chewing activity, and increasing particle size and length of the component, which will in turn increase salivation production and ruminal pH [19]. Also, we would rather control the kind of cereal and avoid using, at the same time, cereals with fast rate of fermentation, thus also avoiding producing a quick drop in the ruminal pH [6]. Therefore, it will be important to take into account not only the grain composition, but also the fermentation rate and grain processing. Rate and extent of starch digestion in the rumen are determined by intricate interrelations among several factors, including source of dietary starch, diet composition, amount of feed consumed per unit time, mechanical alterations (grain processing, chewing), chemical alterations (degree of hydration, gelatinization), and degree of adaptation of ruminal microbiota to the diet. However, almost all the adversities associated with feeding high-grain diets are the result of excessively rapid fermentation of starch. It follows that most feed additives, feed treatments, and management techniques designed to ameliorate these adversities focus on ways to slow the fermentation rate or neutralize the acids produced. Similarly, the main goal of research on grain-processing techniques has been to increase digestibility of grain starch yet to avoid* too much of a good thing* by making starch too readily available for microbial attack [6].

### 6.2. Supplementation with Ruminal Buffers

Another measure could be to incorporate buffers, like sodium and potassium bicarbonate, or alkalinizing agents (sodium and potassium carbonate, magnesium oxide) in the diet, with different objectives, because buffers will be able to neutralize ruminal pH changes; meanwhile the second ones will increase the ruminal pH. They have a direct effect on rumen fluid pH through chemical changes in the rumen because they neutralize acidity through H^+^ sequestration and increase buffering capacity of ruminal fluid, but some experiences have suggested that the potential benefits of controlling ruminal pH with buffers and alkalizers are limited, and they cannot prevent ruminal acidosis alone. This is consistent with the hypothesis that part of the effects observed is pH-independent and should be resolved using alternative feeding strategies [20].

### 6.3. Organic Acids

In the list of feed additives authorized by EU legislation, organic acids fall in the technological group, and their use is currently allowed in all the livestock species. They may be considered safe substances because they produce no detectable abnormal residues in meat.

Organic acids that have been evaluated as feed additives are malic acid, fumaric acid, and aspartic acid. Malic acid and fumaric acid are four-carbon dicarboxylic acids that are found in biological tissues (e.g., plants) as intermediates of the citric acid cycle and are intermediates in the succinate-propionate pathway of ruminal bacteria, such as* Selenomonas ruminantium,* the main gram-negative ruminal bacteria that can account for more than 50% of the total viable bacteria within the rumen [21].

In relation to organic acids and talking about malate, its main characteristics are: (1) stimulation of lactate utilization; (2) increase in ruminal pH, concentrations of propionate, and total volatile fatty acids; (3) increased digestibility of dry matter (DM) and organic matter (OM); neutral detergent fiber (NDF) and hemicellulose; (4) decreased methane production; and (5) decrease in ruminal lactate concentration [6]. But these properties show controversial results in the different* in vitro* and* in vivo* studies. The addition of the acid form to the ration could contribute to reducing buffer blood bases, attributable to the decreased rumen pH, in line with* in vitro* results [22].

### 6.4. Plant Products

It is worth noting that plant bioactivities are still an underexplored area of research and in many cases, although biological activity has been observed, the natural phytochemicals responsible for the activity have not been identified.

In ruminant health, the focus has been on bioactive effect of plants on ruminal flora rather than on specific pathogenic bacteria. This is perhaps understandable, since many of the desirable effects of antibiotics used as growth stimulants act through modification of the ruminal microbe population [23].

In relation to the plant products as feed additives, phytochemical can be classified considering different aspects. So, attending to biological derivation, formulation, chemical description, and purity, phytobiotics comprise a very wide range of substances, and four subclasses in animal feeding may be categorized into (1) herbs (product from flowering, nonwoody, and nonpersistent plants), (2) botanicals (entire or processed parts of a plant, e.g., root, leaves, and bark), (3) essential oils (hydrodistilled extracts of volatile plant compounds), and (4) oleoresins (extracts based on nonaqueous solvents) [24].

Careful selection and combination of these additives may allow the manipulation of rumen microbial fermentation. However, their efficacy requires determination of potential ruminal adaptation in long-term* in vivo* feeding conditions. Thus, results based on short-term* in vitro* fermentation studies with several plant extracts should be interpreted with caution.

### 6.5. Probiotics

Direct-fed microbials (DFM), or probiotics, are live, naturally occurring bacterial supplements that have been used to improve digestive function of livestock.

Feeding bacterial DFM is based on the concept of providing positive postrumen effects on the animal by improving the population of beneficial gut microflora, being able to alter rumen fermentation in order to reduce the risk of ruminal acidosis [25]. The main objective is to stimulate the growing of* Megasphaera elsdenii *(a gram-negative and large coccus which is probably the most important ruminal organism with regard to lactic acid fermentation and, therefore, has a central role in the prevention of ruminal lactic acid accumulation in grain-adapted animals) and/or* Selenomonas ruminantium*, in other words, lactate utilizers bacteria, in order to decrease the risk of ruminal acidosis. So, bacteria (*M. elsdenii* YE34 and* Butyrivibrio fibrisolvens* YE44) could be used to reduce the risk of the process. Lactate-consumers bacteria have also been proposed as DFM and have been used successfully to decrease concentrations of lactate and maintain ruminal pH.* Megasphaera elsdenii* may utilize lactate and prevent drastic pH drops caused by accumulation of lactate in the rumen when fed a highly fermentable diet [26, 27]. Others [25] pointed out that the use of* Megasphaera elsdenii* as a probiotic can reduce lactate accumulation. Inoculating cattle with* M. elsdenii* could be effective in bolstering populations of lactic-utilizers bacteria. Dosing cattle with* M. elsdenii* prior to the introduction of a concentrate diet may successfully prevent the accumulation of lactic acid and resulting acidosis. Yeast (*Saccharomyces cerevisiae*, dried or live-active dry-) and fungi (*Aspergillus oryzae*) have been proposed as alternative to bacterial microbials, with different mode of action. In general, they facilitate beneficial changes in the rumen motility by their stimulation of growth of rumen protozoa [28].

In relation to yeasts, and especially for* S. cerevisiae*, the main effects on rumen fermentation have been stated [28]: (1) increase in rumen pH (+0.03 on average), (2) increase in rumen volatile fatty acid concentration (+2.17 mM on average), with no influence on acetate-to-propionate ratio, (3) decrease in rumen lactic acid concentration (−0.9 mM on average), and (4) increase in total-tract organic matter digestibility (+0.8% on average). Research has indicated an increase in rumen pH or decreased pH depression when yeast culture is included in ruminant diets. However, other studies have found no changes with it. In fact, several reports have shown that dietary composition influences the extent of pH alteration by yeast culture and that ingredients utilized to maintain pH could mask yeast culture's effects [6].

Fungal DFM have been extensively used in ruminants for improving performance and normalizing rumen fermentation, increasing the ruminal bacterial activity and preventing the lactic acid production [25–28].

### 6.6. Immunization

Finally, immunization against ruminal acidosis has been proposed as an alternative to avoid this process. Vaccination against* Streptococcus bovis* and* Lactobacillus* spp. was successful in maintaining greater rumen pH and decreasing L-lactate concentration [29]. Similarly, preparations of polyclonal antibodies against* S. bovis* or* Fusobacterium necrophorum* were successfully applied to calves, reducing rumen concentrations of target bacteria and increasing pH in steers fed high-grain diets [20].

## 7. Conclusion

In the modern beef farming systems, where the main objective is to obtain products of high quality, the concept of quality includes not only a safe product for the consumer, but also the use of farming practices that respect animals' health, especially in intensive systems. It seems that, considering the reviewed studies, animals kept in sustainable conditions, where their production is in line with the physiological processes associated with growth, can provide benefits to the farmers reducing the cost of the treatment. Lactic acidosis is a well-known problem and there are several tools to manage it varying from feed management to immunization. Nevertheless, prevention is the better way, avoiding the imbalance between high intake of nonstructural carbohydrates and low intake of physically effective neutral detergent fiber.

## Figures and Tables

**Figure 3:**
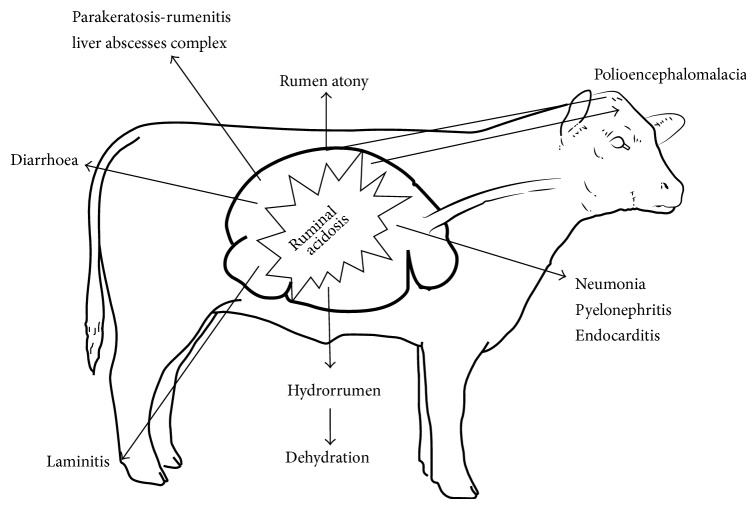
Clinical picture of the disease.

**Table 1:** Main differences between the two different clinical forms of ruminal acidosis [5].

	Ruminal acidosis	
	Acute	Subacute
Presence of clinical signs	Yes	Maybe

Mortality	Yes	No

Ruminal changes		
(1) Rumen pH	Below 5	5.0–5.4
(2) Lactic acid	Increase (50–120 mM)	Normal (0–5 Mm)
(3) Volatile fatty acids (VFA)	Decrease (<100 mM)	Increase (150–225 mM)
(4) Gram negative bacteria	Decrease	Normal
(5) Gram positive bacteria	Increase	Normal
(6) *Streptococcus bovis *	Increase	Normal
(7) *Lactobacillus* spp.	Increase	Normal
(8) Lactic acid producers	Increase	Increase
(9) Lactic acid consumers	Decrease	Increase
Blood parameters		
(1) Blood pH	Low	Borderline
(2) Bicarbonate	Low	Borderline
(3) Lactate	Increase	Normal

**Table 2:** Main causes of ruminal acidosis in feedlots.

High nonstructural carbohydrates intake	
High grain-based diet	
Grain processing (small particles)	
Grain combination of cereals inadequate (type and amount)	
Inadequate ruminal buffers capacity	
Increase in volatile fatty acids (VFA)	
Loss in salivation capacity (including chewing activity)	
Low crude protein in the diet	
Low neutral detergent fiber (NDF)	
Bad bunk management	
Interruptions in normal feed intake patterns	
Nutrition inadequate (including diet changes)	
Stressors	
